# Bibliography

**Published:** 1894-01

**Authors:** 


### Bibliography.

NOTES ON ANÆSTHETICS IN DENTAL SURGERY. By Arthur S. Underwood, M.R.C.S., L.D.S., England, Professor of Dental Surgery, King's College Hospital, etc., and C. Carter Braine, F.R.C.S., Anæsthetist and Instructor in Anæsthetics at Charing Cross Hospital, etc. Second Edition. Claudius Ash & Sons, Limited. London, 1893.

This is the second edition of a very valuable and practical book. It embraces in its one hundred and fifty-five pages the main facts with which it is necessary the young operator should become familiar. These are stated so clearly that no difficulty should be experienced in comprehending the processes detailed.

The author has very wisely not depended entirely upon his own judgment, but has called to his aid specialists in anæsthetics.

The chapters on ether and chloroform in this edition have been entirely rewritten.

The book opens with a short history of anæsthetics, followed by six additional chapters on "General Considerations, etc.," "Nitrous Oxide," "Use of Air with Nitrous Oxide," "Ether," "Chloroform," and "Physiology of Anæsthesia."

The inexperienced operator will find the sub-chapter on "Minor Precautions" to be of special value.

In prolonged operations the use of ether is recommended following the administration of nitrous oxide. The author states that, "in such cases a few respirations of ether administered when the patient is under gas prolongs the anæsthesia, and so small an amount of ether is used that the patient is not aware that he has inhaled anything but nitrous oxide." Then follows a detailed description of the mode of administration.

The use of air in connection with nitrous oxide is described by George Rowell, F.R.C.S., and, after detailing various modes to admit air, says, "The plan which I have adopted is to occasionally interpose a complete breath of air between the breaths of gas by turning the stop-cock of a Hewitt's gas apparatus off and on again before and after an inspiration. . . . When air has thus been carefully given, the resulting anæsthesia is practically as deep and as good as when nitrous oxide alone is employed, and I believe always lengthened."

The chapter on the "Physiology of Anæsthesia" seems worthy of special attention throughout, and especially the closing paragraphs on the danger of shock where many teeth are extracted at one sitting. The theory is that irritation of the fifth pair of nerves is especially liable to be followed by this condition.

We can recommend this book as a safe guide and a valuable work of reference.

Surgery. A Manual for Students and Practitioners. By Bern B. Gallaudet, M.D., Demonstrator of Anatomy and Clinical Lecturer on Surgery, College of Physicians and Surgeons, New York, etc., and Charles N. Dixon Jones, B.S., M.D. Lea Brothers & Company, Philadelphia.

This book of two hundred and ninety-six pages is another of that excellent series of manuals published by Lea Brothers & Co., The care with which these have been prepared is continued in this, and it is satisfactory in the general statements and description of surgical procedures. A considerable portion has been devoted properly to the difficult subject of inflammation. The author has been successful in arranging this to meet the comprehension of the dullest student. His explanation that the exciting cause of inflammation . . . is bacteria may be questioned, in view of the fact that certain inflammations have been demonstrated to have been produced without the agency of micro-organisms. It is doubtful whether dogmatic assertions on this point can be indulged in at this period of the investigations.

This book, as also the others of the series, are valuable as reference manuals, and will, probably be made use of in this direction quite as much as for that for which they were originally intended.

Chemistry and Physics. A Manual for Students and Practitioners. By Joseph Struthers, Ph.B., D. W. Ward, Ph.B., and Charles H. Willmarth, M.S. Edited by Bern B. Gallaudet, M.D. Lea Brothers & Co., Philadelphia.

The series of manuals for students of medicine, published by Lea Brothers & Co., have a well-deserved reputation for the care taken to have each of them regarded as an authority upon the special subject treated.

This the twelfth of the series is of a superior character. The matter is carefully arranged in the form of questions, and the answers given are so tersely stated that they cannot fail to carry with them a clear comprehension of the subject. This must be the impression left upon the mind of reader and student, upon an examination of the admirably-prepared contents of this volume.

The difficulty with students at the commencement of any intricate study is to know how to separate essentials from non-essentials. To accomplish this there is nothing better than the question and answer method, providing these are prepared by practical men.

This book gives evidence of this care, and the fact that three teachers, prominent in their several branches, have labored together in its arrangement is a guarantee for thoroughness in the work and freedom from error.

Annual of the Universal Medical Sciences. A Yearly Report of the Progress of the General Sanitary Sciences throughout the World. Edited by Charles E. Sajous, M.D., and Seventy Associate Editors, assisted by over Two Hundred Corresponding Editors, etc. Illustrated. The F. A. Davis Company, Publishers, Philadelphia, New York, Chicago, and London, 1893.

The sixth issue of this annual is again presented to the medical reading world with a promptness and completeness worthy of the highest praise.

Every issue has been an improvement on those preceding, and this is no exception; indeed, it seems to have nearly reached a culminating point in the particular line of work mapped out for the editors and collaborators. The removal of the editor-in-chief, Dr. Sajous, to Paris, led to the thought that perhaps it might not show that care previously presented, but this is, happily, not the case; indeed, it seems to have given it a broader and strictly more of an international character.

The following eminent names have been added to the active list of contributors, and increases the value of this as it must that of succeeding issues. Among these are Dujardin-Beaumetz, Benjamin Ward Richardson, Lépine, Obersteiner, Bourneville, Kerr, Lutaud, Budin, Buxton, Levison, Apostoli, and Poirier.

The amount of labor represented in these five volumes, containing in brief the yearly work of the world in medical science, is simply enormous, and the wonder is how it could have been accomplished in the limited period of a year. It is the one work of the kind in the world, and it is not, therefore, remarkable that it is fully appreciated wherever it has become known.

